# Burrowing behavior and burrowing energetics of a bioindicator under human disturbance

**DOI:** 10.1002/ece3.5853

**Published:** 2019-11-28

**Authors:** Mustafa R. Gül, Blaine D. Griffen

**Affiliations:** ^1^ School of the Earth, Ocean, and Environment University of South Carolina Columbia SC USA; ^2^ Department of Biology Brigham Young University Provo UT USA

**Keywords:** bioenergetics, ghost crabs, longevity, *Ocypode quadrata*, sandy beach, site fidelity, South Carolina

## Abstract

1. Bioindicator species are extensively used for rapid assessment of ecological changes. Their use commonly focuses on changes in population abundance and individual sizes in response to environmental change. These numerical and demographic shifts likely have behavioral and physiological mechanistic drivers that, if understood, could provide additional insights into the use of these species as bioindicators of habitat health.
2. The Atlantic ghost crab, *Ocypode quadrata*, is a global bioindicator species of human disturbance on sandy shores. Individual size and population abundance of *O. quadrata* decline dramatically at sites with human disturbance, and the causes of this phenomenon remain unclear.
3. Here, we test the hypothesis that individual and population‐level changes at disturbed sites reflect changes in burrowing behavior and energetics. Specifically, we examine whether or not the burrowing behavior (e.g., burrow fidelity and longevity) of *O. quadrata* changes because of human disturbance. We also examine energy required for burrowing by *O. quadrata* across different levels of human disturbance.
4. We show that *O. quadrata* have the highest burrow fidelity and longevity at sites with low level of human impact, and weakest burrow fidelity and longevity at pristine sites. *O. quadrata* reduce the burrowing energy allocation by manipulating the burrow dimension and increasing the burrow longevity even under low levels of human disturbance.
5. Overall, this study shows that human disturbances not only change the behavior of organisms, but also shift energetic balance. Our results support the use of a bioenergetic approach to better understand how human disturbances influence natural populations, and the specific use of this approach with this bioindicator species.

## Graphical abstract

Human disturbance alter the behavior and therefore shift the energetic balance of the organisms.

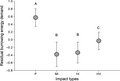

## 1 INTRODUCTION

Bioindicator species are widely used to identify and measure human‐related ecological changes (McGeoch, 1998; Carignan & Villard, 2002; Siddig, Ellison, Ochs, Villar‐Leeman, & Lau, 2016; Spellerberg, 2005) in many different ecosystems from forests (Pearce & Venier, 2006; Rainio & Niemelä, 2003; Maleque, Maeto, & Ishii, 2009) to coral reefs (Erdmann & Caldwell, 1997; Hallock, Lidz, Cockey‐Burkhard, & Donnelly, 2003). The main reason for using bioindicators is the reduced cost, time, and effort compared with examining all biota in a disturbed region (Carignan & Villard, 2002; Cortes, Hughes, Pereira, & Varandas, 2013; Spellerberg, 2005). Thus, the species that is selected as an indicator should represent the ecological changes in a given area (Carignan & Villard, 2002; Siddig et al., 2016), which is particularly important in coastal regions due to the difficulty of assessing the impacts of human disturbances in marine environments (Vitousek, Mooney, Lubchenco, & Melillo, 1997) because of their highly dynamic nature (Carr et al., 2003).

Bioindicator species usually signal changes in a given ecosystem via changes in their presence/absence, abundance, or age/size structure (Carignan & Villard, 2002; Heink & Kowarik, 2010; Siddig et al., 2016; Spellerberg, 2005). However, changes in behavior may also be used as a bioindicator, as species may change their behavior and daily activities under altered conditions due to direct and indirect human disturbances (Sih, Stamps, Yang, McElreath, & Ramenofsky, 2011; Sih, 2013; Wong & Candolin, 2015; Fontúrbel, Candia, Malebrán, Salazar, González‐Browne, & Medel, 2015; Costa, Madureira, & Zalmon, 2019). For example, population abundance of some crab species is commonly used as bioindicators of various human disturbances such as urbanization, mining, and contamination (Cannicci et al., 2009; Jonah, Agbo, Agbeti, Adjei‐Boateng, & Shimba, 2015; Schlacher et al., 2016; Wildsmith et al., 2009), and some of these species additionally alter burrowing behaviors in disturbed sites (Weis & Perlmutter, 1987; Bartolini, Penha‐Lopes, Limbu, Paula, & Cannicci, 2009; Culbertson et al., 2007; Gül & Griffen, 2018a). Species may also alter their feeding habits (Griffiths et al., 2017; Jokimäki, Suhonen, Jokimäki‐Kaisanlahti, & Carbó‐Ramírez, 2016) and trophic interactions (Costa, Tavares, Suciu, Rangel, & Zalmon, 2017; Gray, Baldauf, Mayhew, & Hill, 2007) in areas with human disturbance, and thus these changes in behavior and daily activities can result in changes to the energy balance and physiological state (Chandurvelan, Marsden, Glover, & Gaw, 2015; Spellerberg, 2005). Therefore, despite the fact that studies on the energetic results of human disturbance in bioindicator species are limited to a few examples (Adams & Ham, 2011; Toro, Navarro, & Palma‐Fleming, 2003), there are several examples to show that alterations in the physiological/energetic conditions of nonbioindicator organisms are due to direct (Williams, Lusseau, & Hammond, 2006; Symons, Pirotta, & Lusseau, 2014; Villegas‐Amtmann, Schwarz, Sumich, & Costa, 2015) and indirect human impacts (Griffen, 2018; Leo, Dahlke, Storch, Pörtner, & Mark, 2018; Thomas et al., 2016).

Burrowing behavior is a common phenomenon in organisms (Reichman & Smith, 1990; Lucrezi & Schlacher, 2014; Nomura, Rossa‐Feres, & Langeani, 2009). Despite its energetically expensive nature (Hunter & Elder, 1989), burrowing behavior provides some advantages, including protection from predators and cannibals, from direct human disturbance, and from harsh environmental conditions (Soriguer & Amat, 1980; Christoffers, 1986; Friend, 1993; Gül & Griffen, 2018a; Lucrezi & Schlacher, 2014). Burrowing species commonly use olfactory and visual cues to return to their burrows (Hughes, 1966; Bonadonna, Spaggiari, & Weimerskirch, 2001; Ribeiro, Christy, Rissanen, & Kim, 2006; Lucrezi & Schlacher, 2014) and as a result, show high fidelity to individual burrows and sites.

Intensity of human disturbance on sandy beaches generally increases with human population size (Davenport & Davenport, 2006; Defeo et al., 2009; Halpern et al., 2008). The ecological impacts of this disturbance are commonly assessed using the population abundance and size structures of common macroinvertebrates such as clams (Defeo & de Alava, 1995; Schlacher, Thompson, & Walker, 2008; Sheppard, Pitt, & Schlacher, 2009), mole crabs and sand hoppers (Cardoso, Barboza, Skinner, & Cabrini, 2016), beetles (González, Yáñez‐Navea, & Muñoz, 2014), and ghost crabs (Aheto, Asare, Mensah, & Aggrey‐Fynn, 2011; Barros, 2001; Gül & Griffen, 2018a, 2018; Hobbs, Landry, & Perry, 2008; Lucrezi, Schlacher, & Robinson, 2009; Neves & Bemvenuti, 2006; Schlacher et al., 2016; Steiner & Leatherman, 1981; Wolcott & Wolcott, 1984).

The most widespread responses of ghost crabs to human disturbances on sandy shores are decreases in population abundance and individual body sizes (Schlacher et al., 2016 and citations therein; Gül & Griffen, 2018a, 2018). These changes are often measured using burrow counts, a nondestructive and efficient technique for assessing both the abundance and size distribution of ghost crabs (Schlacher et al., 2016). Various mechanisms have been hypothesized for the reduced density and size of ghost crabs with human disturbance, including higher mortality rate for individuals with shallow burrows (Schlacher, Thompson, & Price, 2007), direct crushing by vehicles (Wolcott & Wolcott, 1984), lower organic material availability (Stelling‐Wood, Clark, & Poore, 2016), and stress due to direct handling by people (Gül & Griffen, 2018a). However, the precise mechanistic reason for observed demographic changes remains unclear. Besides these demographic changes, ghost crabs also alter their burrow architecture under human influence (Gül & Griffen, 2018a; Lucrezi & Schlacher, 2010; Schlacher & Lucrezi, 2010), implying that there may be an energetic component to the response of ghost crabs to disturbance. Here, we investigate whether Atlantic ghost crabs, *Ocypode quadrata*, show any variation in the burrowing behavior (fidelity and longevity) and energy demand for burrowing activities between sites with different levels of human disturbance. We predicted that *O. quadrata* will be forced to burrow more frequently on disturbed sites because their burrows would be destroyed by human activities more often compared with pristine sites. We further predicted that burrowing would represent an energetically expensive behavior and that this energetic cost should be influenced by changes in the frequency of burrows and the size of burrows across beaches with different levels of human disturbance.

## 2 MATERIALS AND METHODS

### 2.1 Study system

We examined the burrowing behavior (e.g., burrow fidelity and longevity) of the Atlantic ghost crab, *O. quadrata*, on twenty South Carolina sandy beaches with different levels of human disturbances between 15th May and 1st November 2017 (Table 1). We classified and grouped our study sites by using the urbanization index (UI) that was modified from González et al. (2014). Specifically, we used the following six variables based on observations and counts during the summer of 2016 and 2017 to estimate urbanization levels: (1) proximity to urban centers, (2) building on the sand and dunes, (3) beach cleaning, (4) number of vehicles on the sand, (5) visitor frequency, and (6) infrastructure such as parking lots, restrooms, and other amenities. Variables 3, 4, and 5 were obtained by direct counts during our study. Since beach cleaning is performed during the nights, we observed whether the beach was mechanically cleaned and counted the number of vehicles on the beach during the night. For frequency of visitors, we counted all visitors for two hours in the morning between 09:00 and 11:00 as a proxy. On the other hand, levels 1, 2, and 6 were acquired by direct observations. We scored each level from “0” to “5” based on the level of estimated variables (Table S1, Appendix S1). We then summed these scores across the six variables described above and divided by 30, providing a UI score that ranged from 0 to 1. Finally, we grouped our study sites by using UI scores as pristine (P: from 0 to 0.25), moderately impacted by only visitors (MI: from 0.26 to 0.50), highly impacted by only visitors (HI: from 0.51 to 0.75), and highly impacted by human visitors and vehicles (HV: from 0.76 to 1). While this UI score is a continuous variable, we bin it as described here for ease of presentation and, more importantly, to avoid over representing the certainty of a specific level of human impact at each site. Ultimately, the UI scores were determined for each site at a single point in time and therefore only represent approximations of the level of human disturbance at each site.

**Table 1:** Levels of the urbanization indicators from absent (0) to extremely high (5) level and the urbanization index (UI) of the study sites

No	Site	Proximity to urban centers	Building on the sand and dunes	Beach cleaning	Number of veh. on the sand	Frequency of visitors	Infrastructures	UI
1	Waties Island 1	0	0	0	0	1	0	0.03
2	Waties Island 2	0	0	0	0	1	0	0.03
3	Waties Island 3	0	0	0	0	1	0	0.03
4	N. Myrtle Beach 1	5	4	5	4	5	5	0.93
5	N. Myrtle Beach 2	5	4	5	4	5	5	0.93
6	Myrtle Beach 1	5	4	5	5	5	5	0.96
7	Myrtle Beach 2	5	4	5	5	5	5	0.96
8	Garden City Beach	5	5	5	3	5	4	0.9
9	Pawley's Island 1	3	4	0	0	2	2	0.36
10	Pawley's Island 2	3	4	0	0	2	2	0.36
11	Debidue Island 1	1	0	0	0	1	0	0.06
12	Debidue Island 2	1	0	0	0	1	0	0.06
13	Isle of Palm 2–1	5	4	3	2	4	3	0.7
14	Isle of Palm 2–2	5	4	3	2	4	3	0.7
15	Isle of Palm 1–1	3	3	0	0	2	1	0.3
16	Isle of Palm 1–2	3	3	0	0	2	1	0.3
17	Sullivan's Island 1	4	3	3	3	4	3	0.66
18	Sullivan's Island 2	4	3	3	3	4	3	0.66
19	Folly Beach	4	4	1	0	3	3	0.5
20	Burkes Beach	4	2	2	2	5	5	0.66

Note

The sites were aligned based on their latitudes from north to south.

### 2.2 Impact of marking procedure

We used a mark–recapture study to examine burrow fidelity (described below). Prior to this, we used a preliminary study to determine whether our mark and recapture technique would likely alter burrow fidelity and longevity of ghost crabs. This preliminary experiment did not determine whether our procedure influenced the burrow fidelity of crabs. We therefore make the assumption that any impacts of our procedures on burrow fidelity did not differ across levels of human impact. We randomly selected one site for each of the four human disturbance levels. At each site, we selected newly created burrows in the upper zone (i.e., on the seaward side of the dune vegetation) and designated them as control or treatment burrows. Burrow was identified as “newly‐created” by initially marking existing burrows and selecting new burrows that appeared beginning the next day for the study as they were created by crabs. We examined a total of 84 burrows (i.e., 42 control, 42 treatment) across the four sites. Each burrow was marked using a marking flag. We were forced to burry our marking flags in the sand at about a 20‐cm depth in the sites with human presence because beachgoers removed unburied flags. The experimental controls consisted of a set of burrows used by crabs that had not been marked. For these control burrows, we used a set of burrows that were newly created over a 3‐day period and examined these until they collapsed to determine the burrowing behavior and to measure burrow longevity. The experimental treatment consisted of a set of burrows used by crabs that had been marked for identification. For these treatment burrows, we set up simple traps (approximately 45 cm deep buckets buried flush with the sand and with a piece of rotting fish meat to lure crabs into the buckets) before sunset to capture the crabs that newly created their burrows. We also surrounded burrows and traps together using approximately 30 cm high plastic mesh to guide crabs into the traps. We were careful not to destroy the sand mounds and not to remove any material (e.g., small stones) from around the burrows, as individuals use sand mounds (Hughes, 1966; Lucrezi & Schlacher, 2014) and marine debris (Costa et al., 2019) as visual cues to recognize their own burrow. All captured crabs in the buckets were marked on the ventral side using nail polish, which is visible for up to 2 months in this species (Christoffers, 1986). After we completed our marking procedure, we removed the traps and mesh around the burrows. We applied the same marking procedure for all newly created burrows and crabs for the next 3 days. To ensure that an individual still owned the same burrow, we then applied the same trapping technique every 3 days until the burrow collapsed. It is possible that crabs escaped our capture techniques and were therefore present, but not sampled. Thus, or estimates of burrow fidelity here are conservative estimates (i.e., they could potentially overestimate the possible impacts of our marking procedures on burrow fidelity). We analyzed the results of this preliminary experiment using a generalized linear mixed‐effects model (GLMM) with a Poisson distribution to understand whether the number of days during which the burrows still existed (i.e., burrow longevity) varied between burrows in the control (unmarked crabs) and treatment (marked crabs) groups. We treated the experimental group (control vs. treatment) and the level of human impact as fixed factors, with site treated as a random factor to control for multiple samples at each site. We used the statistical software R version 3.5.1 (R Core Team, 2018) for this and all subsequent statistical analysis in this paper.

### 2.3 Use of foreign burrows

We conducted a second preliminary experiment to determine whether crabs would use a burrow that they did not construct. If crabs will use a burrow that they did not construct, then they may avoid the energetic cost of burrow construction. During our study, we recognized that marked individuals returned to their own burrows immediately after we had released them. We also observed that foraging individuals run back to burrows from a certain distance (i.e., up to approximately 8–10 m) when they encounter humans. Thus, to test whether *O. quadrata* will enter any random burrow, we collected 10 individuals of various body sizes on Waties Island (one of our pristine sites). We kept these individuals in separate plastic containers for about 10 min, during which time we shook the containers gently to disturb them. We then chose random burrows that were matched to the size of individual crabs and released the individuals, one by one, next to the mouths of these burrows. We conducted this experiment at night when crabs are usually out of their burrows foraging, reducing the likelihood that these burrows were already occupied by their resident crab. When an individual did not immediately try to escape or enter the burrow, we pretended to catch them to elicit a response. We applied a Chi‐squared test to determine whether crabs entered random burrows. We only conducted this experiment at one of our pristine sites and therefore cannot be sure that the behavior does not differ with the level of human impact.

### 2.4 Burrow fidelity

To understand whether the number of days during which an individual uses the same burrow (burrow fidelity) varies based on the level of human disturbances, we applied a mark and recapture technique as described above at one site for each disturbance level. For the burrow fidelity, we sampled the newly created burrows over a 1‐week period in three replicate rectangular quadrats (10 × 5 m) situated on the seaward side of the dune vegetation at each site. We established our quadrats near the dune vegetation because the persistence of ghost crab burrows is directly affected by the tides (Evans, Cram, Eaton, Torrance, & Wood, 1976) and height on the beach (Campagnoli, Pombo, & Turra, 2018). We first measured burrow size as the largest distance across the burrow openings, which is positively related to crab size (*r* ^2^ = 0.98, Wolcott, 1978; Silva & Calado, 2013; de Oliveira, Souza, & Soares‐Gomes, 2016; Souza, Oliveira, Tardem, & Soares‐Gomes, 2017). Then, the same trapping procedure explained above was repeated every 3 days. During our investigation, we marked individual crabs and their burrows with the same numbers so that individual crabs and their burrows could be matched. Since some individuals destroyed their own burrows after they were disturbed, we observed the sampled burrows after a couple hours to see whether the burrows still existed. If not, we excluded that individual burrow and the crab from our data. We used a generalized linear mixed‐effects model (GLMM) with a Poisson distribution to test whether burrow fidelity (number of days a burrow was used) varied among the levels of human impacts and the size of the burrow (fixed factors). To control for latitudinal and temporal difference, sampling day (e.g., Julian days) and latitude were included as random factors in this, and all subsequent mixed models described below (Fox & Weisberg, 2018; Galwey, 2014). Following this analysis, we applied a Tukey's HSD test to make multiple pairwise comparisons of burrow fidelity among the levels of human impact.

### 2.5 Burrow longevity

To examine whether the persistence of the ghost crab burrows themselves was influenced by the level of human impact, we observed the longevity of *O. quadrata* burrows (i.e., the number of days from creation until collapse) in three trial rectangular quadrats (20 × 10 m) in each site listed in Table 1. We established quadrats on the seaward side of the dune vegetation. We marked the burrows in each quadrat using orange marking flags. We then marked all subsequent newly created burrows in each site with blue or green flags for the following week. We observed the burrows marked with blue and green flags every day until they collapsed. To determine whether burrow longevity varied among the levels of human impact or burrow size (fixed factors), we ran a generalized mixed‐effects model (GLMM) with a Poisson distribution, followed by a Tukey's HSD test for multiple comparisons between levels of human impacts.

Geomorphological characteristics of sandy beaches and burrow densities at each site were determined as possible explanatory variables of the burrowing behavior (details in Appendix S2). We ran two generalized linear mixed‐effects models (GLMM) with Poisson distributions to determine whether burrow fidelity and longevity were influenced by crab density, sand grain size, and sand compaction (fixed variables).

### 2.6 Energy requirement of burrowing

We conducted an experiment to determine the energetic coast of digging for *O. quadrata*. We collected 40 individuals of a range of sizes in both sexes from Isle of Palm in September 2017. These were transported to the University of South Carolina in Columbia, South Carolina where they were held in separate plastic containers (length 23.1 cm, width 16.2 cm, and height 13.9 cm) with ~5 cm moist sand. We weighed them and kept them without food for 3 days to standardize their hunger level, after which we fed them every other day with commercially purchased salmon for next 10 days. Each individual was offered 10% of its wet body weight at each feeding, and uneaten food was removed after 24 hr. All of this was done in an attempt to standardize the energetic conditions across crabs.

Following this holding period, we transported the crabs back to the Isle of Palm where they were collected. We created experimental chambers by excavating 20 pits in the sand at Isle of Palm on the beach berm. This was done at night to avoid changing the temperature of sand at depth via direct sunlight. Into each pit, we placed a 121 L plastic can (diameter 55.8 cm, height 81.2 cm) with approx. 50 small holes drilled into the sides and the bottom to allow moisture and temperature exchange between the inside of the chamber and the surrounding sediment. We then replaced the sand that had been removed into these experimental chambers. To mimic the sand compaction of the surrounding beach, we excavated another identical pit and measured the sand compaction every ~20 cm during excavation using a pocket penetrometer. Then, as the chambers were filled with sand, we measured the compaction inside the chambers at each 20‐cm interval and pressed the sand as necessary to achieve the same compaction level as was observed in the surrounding sediment. We surrounded the mouth of the chambers with a vertical cardboard barrier to keep the crabs from escaping and then allowed 4 hr for moister and oxygen levels in the chambers to equilibrate. We then released an individual treatment crab onto the sand surface in each chamber and allowed them to excavate burrows. During this time, we held the other 20 control crabs in their transport containers under the same ambient environmental conditions with the treatment crabs in the field. After about 2 hr, all experimental crabs had excavated a burrow. To force the individuals to leave their burrows, we used a smoke fumigator (Pombo & Turra, 2013). Twelve of the crabs exited their burrows due to the smoke, while the others remained in their burrows until we poured plaster of Paris into the burrow. We determined the burrow volume using the burrow casts created with plaster (see Appendix S3). We removed the second digging leg (i.e., the walking leg on the same side as the minor claw (Lucrezi & Schlacher, 2014)) from each treatment and control crab and immediately placed these in individually labeled plastic bags on ice.

We used the glycogen content in the digging leg muscle tissue as a proxy of the energetic cost of digging. To determine the glycogen content, we used a Sigma‐Aldrich Glycogen Assay Kit MAK016. We used the instructions provided by the manufacturer of the kit to perform the glycogen analysis on a subsample (~10 mg) from each leg muscle removed from the experimental and control crabs. Finally, we measured absorbance of our samples under a spectrophotometer (Biotek Synergy H1 Hybrid Reader working with software Gen5). The glycogen content of the samples was calculated from a standard curve [glycogen content (μg/mg of muscle tissue) = 1.9837 × OD(570 nm) − 0.0269; R^2^ = 0.9978] that we constructed simultaneously with our glycogen samples. To determine the energy level in the muscle tissues, we converted the glycogen content to energy by multiplying by 17.2 kJ/g (Lucas, 1996). Our visual inspection of graphs suggested that glycogen content in the leg tissue of experimental crabs declined nonlinearly with burrow volume. We compared three models using AIC to determine the best model to explain this relationship (Burnham & Anderson, 2002). Specifically, we fit a linear model, a negative exponential model, and a quadratic model. We also included crab size in each of these models, reasoning that it would require less energy for a larger crab than for a smaller crab to create a given size burrow. We also used a *t* test to compare the energy level in the muscle tissues between experimental crabs that burrowed and control crabs that did not.

Lastly, we estimated the annual energy demand required to support the burrowing behavior of *O. quadrata*. We did this by combining our data on burrow longevity at each of our sites and the energy expended during the removal of 1 cm^3^ of sand (calculated from the energetic costs described above), with overall burrow volume that we have previously measured at these same sites (Gül & Griffen, 2018a), and with the portion of the year on the Atlantic coast when crabs actively burrow (known to extend from April to November on the Maryland coasts, Christoffers, 1986). This is admittedly a rough estimate, since it assumes that energetic costs do not change seasonally, and also does not include the costs of maintaining the burrow once it is dug. Because the burrow volume is correlated with the crab size (Chan, Chan, & Leung, 2006), we removed the effects of crab size by regressing the log‐transformed calculated burrowing energy demand against log‐transformed burrow opening diameter (Packard & Boardman, 1999). The standardized residuals obtained from this regression were used as a response variable for a 1‐way ANOVA to compare this estimated size‐independent annual energy demand for burrowing in ghost crabs on beaches with different levels of human disturbance. Before this statistical analysis, we conducted a Shapiro–Wilk test of normality and Levene's test for the equality of variances to assess whether the data fit the assumptions of parametric tests. ANOVA was followed by a Tukey's HSD test for multiple comparisons among groups.

## 3 RESULTS

### 3.1 Impact of marking procedure

The trapping and marking technique used here to determine the burrow fidelity did not seem to alter the longevity of the burrows. The burrow longevity in the treatment group was slightly lower than in the control group under each level of human impact; however, none of these differences were significant (GLMM, *p* > .5 for the overall comparison of burrow longevity for marked and unmarked crabs as well as for the interaction term between control/treatment and each level of human impact). Thus, we conclude that any artifact introduced by our capture and marking procedures was minimal.

### 3.2 Use of foreign burrows

We did not find any instances of a foreign crab occupying one of our focal burrows during any part of our study. This is also consistent with *O. quadrata* being reluctant to enter burrows that were not their own in our small experiment where we tried to elicit foreign burrow use (Chi‐squared test,* X* ^2^ = 6.4, *df* = 2, *p* = .0011). Specifically, we observed that 9 out of 10 individuals did not enter the foreign burrow that they were released next to, even though that burrow was large enough for them. Only one individual entered shallowly into the foreign burrow, staying very close to the entrance, and when we disturbed it using a thin stick, it left the burrow immediately and ran away rather than receding further into the burrow.

### 3.3 Burrow fidelity

**Figure 1:**
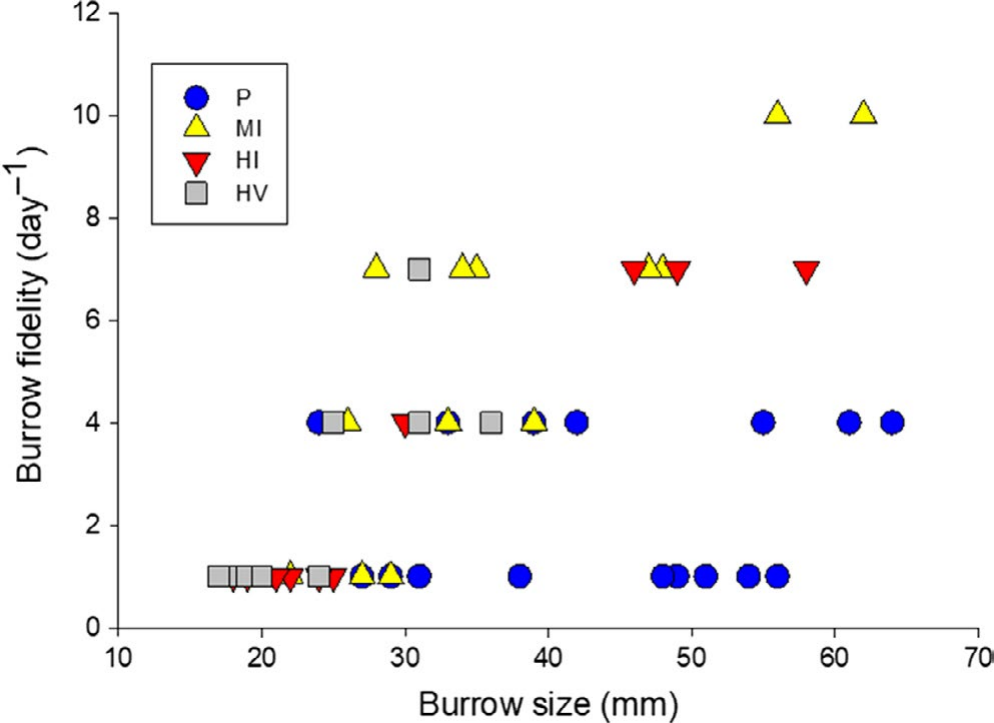
The relationship between the burrow fidelity and the burrow opening diameter at the sites with various levels of human disturbance. Burrow fidelities were observed in Waties Island (pristine site), Pawley's Island (moderately impacted site), Isle of Palm (highly impacted site by people), and Garden City Beach (highly impacted site by people and vehicles)

The average burrow fidelity of *O. quadrata* was significantly lower in pristine sites (average = 2.31 ± 0.38 day^−1^, range: 1–4 day^−1^, *n* = 16) compared with burrow fidelity in moderately impacted sites (average = 5.38 ± 0.87 day^−1^, range: 1–10 day^−1^, *n* = 13; GLMM, *Z* = 5.18, *p* < .001), highly impacted sites by people (average = 3.1 ± 0.9 day^−1^, range: 1–7 day^−1^, *n* = 10; GLMM, *Z* = 2.8, *p* = .005), and highly impacted sites by people and vehicles (average = 2.66 ± 0.72 day^−1^, range: 1–7 day^−1^, *n* = 9; GLMM, *Z* = 2.99, *p* = .002, Figure 1). We further found that larger individuals had a stronger burrow fidelity when impact levels were pooled (GLMM, *Z* = 5.62, *p* < .001, Figure 1). Burrow fidelity in *O. quadrata* was negatively impacted by burrow density (GLMM, *Z* = −4.419, *p* < .001) and sand compaction (GLMM, *Z* = −3.152, *p* = .0016). No impact of sand grain size on burrow fidelity was detected (GLMM, *Z* = −1.556, *p* = .11). No significant impact of the interaction terms was detected.

### 3.4 Burrow longevity

**Figure 2:**
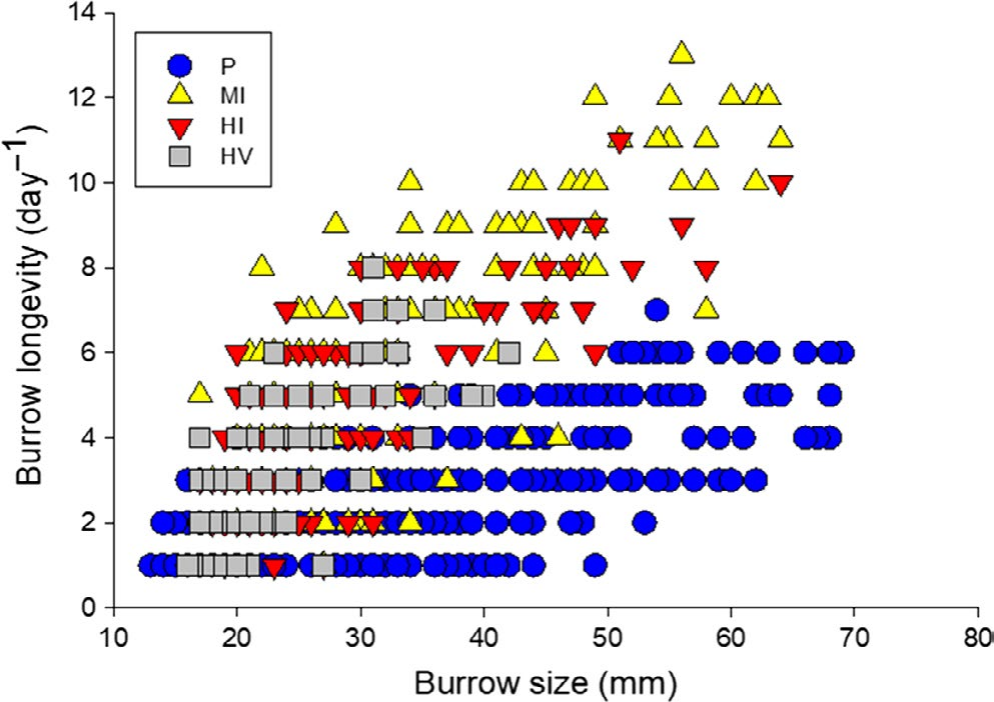
The relationship between the burrow longevity and the burrow opening diameter at sites with various levels of human disturbance. Burrow longevities were observed daily at all sites. Study sites the same as listed in Table 1

Burrow longevity in pristine sites (average = 2.77 ± 0.08 day^−1^, range = 1–6 day^−1^, *n* = 340) and in highly impacted sites by people and vehicles (average = 3.49 ± 0.19 day^−1^, range = 1–8 day^−1^, *n* = 77) was similar (GLMM, *Z* = −0.26, *p* = .78). Contrary to this, a greater burrow longevity was observed in moderately impacted sites (average = 6.71 ± 0.26 day^−1^, range = 1–13 day^−1^, *n* = 132; GLMM, *Z* = 5.74, *p* < .001) and in highly impacted sites by people (average = 4.91 ± 0.22 day^−1^, range = 1–11 day^−1^, *n* = 105; GLMM, *Z* = 3.66, *p* < .001) compared with pristine sites (Figure 2). Also, larger burrows persisted longer than smaller ones (GLMM, *Z* = 11.51, *p* < .001).

There were significant interactions between burrow density and sand grain size (LM, *t* = −3.485, *p* = .0005), between burrow density and sand compaction rate (LM, *t* = −11.63, *p* < .001) and between sand compaction rate and the sand grain size (LM, *t* = 4.288, *p* < .001). These interactions influenced burrow longevity (Table 2).

**Table 2:** Results of generalized linear mixed model (GLMM) testing the effects of fixed factors on the burrow longevity of *Ocypode quadrata*

Fixed factors	*SE*	*Z*‐value	*p‐*value
*A* (burrow density)	1.305	−10.305	<.001*
*B* (grain size)	3.331	−6.15	<.001*
*C* (sand compaction)	7.294	−7.07	<.001*
*A* × *B*	4.325	9.076	<.001*
*B* × *C*	14.262	10.046	<.001
*A* × *B* × *C*	49.093	−8.331	<.001*

Random factors	Variance component	*SE*
Latitude	<0.001	0.00014
Julian days	<0.001	0.1412

Note

Latitudes of the study sites and sampling days (as Julian days) were included as random effects.

* The significant values.

### 3.5 Energy requirement of burrowing

**Figure 3:**
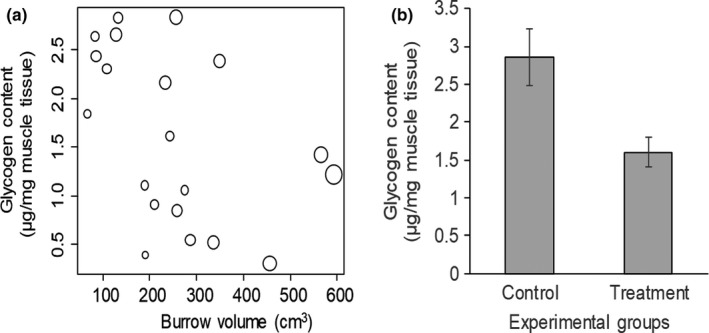
The relationship between the burrow volume and the glycogen content in the second digging leg tissue of treatment crabs (*n* = 20), *Ocypode quadrata*. *Relative circle size indicates crab carapace width* (a). Variation in the glycogen content in the second digging leg tissue of the treatment and the experiment crabs, error bars indicate standard errors (b). Burrowing experiment was conducted on Isle of Palm in 2017

We found that the linear model provided the best fit to explain the relationship between glycogen content in the leg tissue and the burrow volume, but that the fit of this model was statistically indistinguishable from the fit of the quadratic model (AIC value for linear model = 40.79, AIC value for exponential model = 46.21, and AIC value for quadratic model = 41.92). The glycogen content in the digging leg tissues of the individuals in the treatment group increased with crab size by 0.123 ± 0.031 μg for every mm increase in carapace width of the crab (*t* = 3.96, *p* = .001, Figure 3a) and decreased by 0.007 ± 0.001 μg with each additional cm^3^ of burrow volume created (*t* = −4.91, *p* = .0001, Figure 3a). Consequently, the glycogen content in the digging leg tissues of the individuals in the treatment group declined by 78% on average compared the individuals in the control group (*t* test, *t* = 2.96, *df* = 28.625, *p* = .0062, Figure 3b).

**Figure 4:**
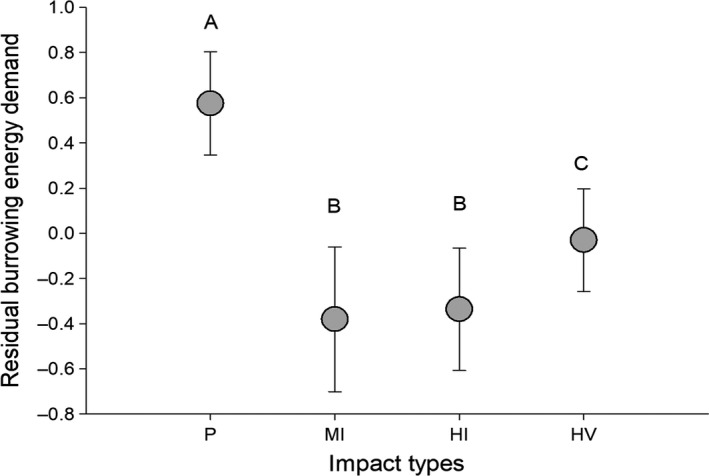
Variation in residual calculated annual burrowing energy demand (mean ± *SD*) of *Ocypode quadrata* under various levels of human disturbance. Letters within graph represent significant differences

Ghost crabs spend 85.525 J of energy to remove 1 cm^3^ of sand during our experiment. Using this value as described in Section 2, we found that the calculated (conservative) annual energy demand of burrowing in ghost crabs was significantly lower for all levels of human disturbance compared with pristine sites (ANOVA, *F* = 116.8, *df* = 3, *p* < .0001, Figure 4).

## 4 DISCUSSION

We have shown that *O. quadrata* alter their burrowing behavior (e.g., burrow fidelity and longevity) under the influence of various levels of human disturbances. We have also demonstrated that *O. quadrata* spend a much higher amount of energy in pristine sites for their burrowing behavior compared with the sites disturbed by people. We have also shown that larger individuals generally return to the same burrow for a longer period of time compared with smaller individuals. Since burrows protect crabs from predators, cannibals, and environmental influences such as strong winds, cold, and hot weather (Christoffers, 1986; Lucrezi & Schlacher, 2014) and desiccation (Antia, 1989; Gül & Griffen, 2018a), changing the energetics of this behavior is likely to shift the ecology, physiology, and demographics of *O. quadrata*. These results have important implications for this system and for our understanding of ecological changes due to human disturbance more broadly.

### 4.1 Implications for this system

Burrow fidelity and longevity increased at sites with moderate human disturbance compared with heavily disturbed and pristine sites. Specifically, individuals used the same burrows for up to 10 days at sites with moderate human disturbance. Pristine sites on the coast of South Carolina have the highest ghost crab abundance and the lowest sand compaction rates (Gül & Griffen, 2018a, 2018; Table S4). However, while an inverse relationship between population density and the duration of burrow use has been reported (Hughes, 1966), the sand compaction rate may be the main mechanism that determines the length of the burrow persistence, as suggested by the positive relationship between sand compaction and burrow longevity reported here.

The positive relationship between the size of the individuals and the length of time during which a burrow is used likely reflects the location of burrows of different sizes on the shore. Larger burrows are found much closer to the back shore on South Carolina beaches, while smaller crabs are outcompeted on the upper shore and are relegated to digging their burrows lower down on the shore (Gül & Griffen, 2018). An inverse correlation between tidal height and burrow persistence exists (Costa et al., 2019; Evans et al., 1976; Hughes, 1966), likely due simply to increased frequency of inundation by waves lower down on the shore. We attempted to control for effects of tidal influence by examining only burrows at the same relative tidal height at all sites; however, our transects were wide enough (e.g., 10 m) to contain small burrows at the lower edges of the transects. Alternatively, the visual cues such as mounds and debris, which are used by *O. quadrata* to recognize their burrows (Costa et al., 2019; Hughes, 1966; Lucrezi & Schlacher, 2014), may be removed more frequently around the smaller burrows due to their relative lower location on the shore.

We found that the glycogen level in the leg tissues dropped by almost 50% after 2 hr of burrowing. Assuming that this energy required to initially dig a burrow is greater than the energy required to maintain a burrow, the longevity of burrow use and the energy allocated for digging annually should be inversely related to each other, and this relationship is influenced by human disturbance in *O. quadrata* populations. Specifically, we calculated that ghost crab living in pristine sites has the highest burrowing energy demand, while individuals living under human disturbance decrease the amount of annual energy requirements for burrowing by creating smaller and simpler burrows (Gül & Griffen, 2018a; Lucrezi & Schlacher, 2010; Schlacher & Lucrezi, 2010) and by increasing their burrow fidelity and longevity. These reductions in energy allocation to burrowing may be required if *O. quadrata* gain less energy through foraging on beaches that are more highly disturbed, as human activities also negatively impact common ghost crab prey such as bean clams (*Donax* spp.), mole crabs (*Emerita* spp.), and sandy beach coleoptera (*Phaleria* spp.) (Cardoso et al., 2016; González et al., 2014; Schlacher et al., 2008; Sheppard et al., 2009).

Finally, we examined only the amount of energy that is allocated by individuals based on their initial burrowing behaviors. However, crabs will also need to expend energy to maintain their burrows, and these daily maintenance costs should also differ across beaches based on human impacts because of the influence of humans on sand compaction. However, our findings that burrow longevity only slightly exceed burrow fidelity (i.e., burrows collapse after they are left vacant for ~1 day) suggests that burrow maintenance is considerable. Further research is needed to examine the relative costs of initial burrow creation and burrow maintenance, as well as seasonal variation since crabs will demand more energy during the reproduction period.

### 4.2 Broader implications for ecology

Our study has at least two broader implications for the population ecology of species used as bioindicators of human disturbances. First, in agreement with countless other studies, our study shows that species alter their behaviors and daily activities, and therefore their energetic balance, in the presence of human disturbance. Despite the fact that lower population density and smaller individual sizes are widely used as common responses of bioindicator species to human disturbances (Carignan & Villard, 2002; Heink & Kowarik, 2010; Siddig et al., 2016; Spellerberg, 2005), the mechanistic reasons for those demographic changes are often not well understood. Further, while many studies have examined the behavioral and energetic impacts of indirect human disturbances including climate change and contamination (Bonnard, Romeo, & Amiard‐Triquet, 2009; Griffen, 2018; Schmidt et al., 2017; Williams et al., 2006), relatively few studies have focused on the energetic consequences of direct human impacts (e.g., tourism, coastal reclamation etc.) on bioindicator species (but see Adams & Ham, 2011; Toro et al., 2003). Our results highlight that focusing on behavioral changes and their energetic consequences may elucidate the mechanistic reasons behind the declines in individual sizes, because species may exhibit tradeoffs between their growth rate and daily activities that are crucial for their survival.

Second, the link between the levels of human disturbance and energy allocation suggests that this mechanistic link may improve the power to predict the impacts of human disturbances. While examining changes in abundance and individual body sizes within populations is a quick and cost‐effective technique for assessing the extent of human disturbance (Carignan & Villard, 2002; Cortes et al., 2013; Spellerberg, 2005), documenting these demographic changes does not provide any understanding regarding the mechanism(s) behind these responses. To go beyond documenting existing trends and to make predictions about responses to future conditions, ecologists need mechanistic approaches that are applicable to various species under a variety of disturbance types and in different systems. This is especially urgent given the increasing frequency and strength of anthropogenic disturbance as human population sizes increase (Davenport & Davenport, 2006; Halpern et al., 2008; Vitousek et al., 1997). Previous work has argued that understanding the physiological and energetic state of organisms can provide a level of mechanistic predictive power for forecasting future conditions (Pörtner & Farrell, 2008). Our study supports this idea and demonstrates how a bioenergetics approach may be used to explore the consequences of behavioral changes that accompany habitat disturbance by humans.

## CONFLICT OF INTEREST

None declared.

## AUTHORS CONTRIBUTIONS

MRG and BDG conceived and designed the experiments. MRG performed the experiments. MRG and BDG analyzed the data and wrote the manuscript.

## Data Availability

All data used in this study are available from the Dryad Digital Repository: https://doi.org/10.5061/dryad.4b8gtht7z
