# Supplementary material for: Burrowing behavior and burrowing energetics of a bioindicator under human disturbance
Source: Ecol Evol. 2019 Nov 28;9(24):14205–16. doi: 10.1002/ece3.5853 (PMC6953561; doi:10.1002/ece3.5853)
Supplement: Supplementary file 1 [file ECE3-9-14205-s001.docx]

**Burrowing behavior and burrowing energetics of a bioindicator under human disturbance**

**Mustafa R. Gül and Blaine D. Griffen**

**Supplementary Material**

**Appendices 1- 5**

**Appendix 1:**

Variables used to estimate the UI scores

(*Table S1*)

**Appendix 2:**

Assessing geomorphological characteristics and burrow density of sandy beaches

**Appendix 3:**

Analysis of burrow volume

**Appendix 4:**

Geomorphological characteristics and burrow density

(*Table S4*)

**Appendix 1:** Variables used to estimate the UI scores

**Table S1.** Levels and score used to estimate UI for South Carolina sandy beaches (modified from González et al., 2014).

| Scores | Proximity to urban centers | Buildings on the sand and dunes | Beach cleaning  the sand | Number of vehicles on | Visitor frequency | Infrastructure |
| --- | --- | --- | --- | --- | --- | --- |
| 0 | Totally rural. Kilometers away from city center. No access by car. | No building close to beach | Never cleaned mechanically | No access to the beach by vehicle. | No visitor at all. | No infrastructure. |
| 1 | Totally rural. A few kilometers away from city center. No access by car. Pathway exists | Building close to beach about a few kilometers. | Beach mechanically cleaned if needed (may be once a while) | No vehicle and tire traces observed. | Almost no visitor. Pathway exists. | Only pathway and very limited access. |
| 2 | Located close to urban center. Some impact of urban center like noise or light | Buildings exist but far away | Beach mechanically cleaned a few times in a year. May be once a month. | Access by vehicle is very limited. Some irregular traces observed. | Scarce visitor. | Only parking lot. |
| 3 | Close to urban center. Accessed by car. Vehicles pass nearby. | Buildings close to the beach but not on the dunes or sand. | Beach mechanically cleaned more frequent than once a month. | Moderately vehicle access. Some irregular traces. | Mostly local visitors. Has a public access. | Parking lot and some amenities. Good access. |
| 4 | Next to the urban center. Direct access by car. Direct impact by noise and light. | Building just next to the beach, but not on the dunes or sand. | Beach continuously mechanically cleaned. May be a few times a week. | Easily vehicle access. Dense traces. Vehicles observed. | Public access. High visitor demand. | Good access. Good infrastructure. |
| 5 | The city is next to the beach. Direct access by car and direct heavy impact by light and noise. | There are building on the dunes and/or sand. | Beach mechanically cleaned every day. | Advance access to the beach by vehicle. Different types of vehicles observed. | Public access. Very high visitor demand. Destination of long vacations. | Very good access and infrastructure including security and life guards. |

**Appendix 2:** Assessing geomorphological characteristics of sandy beaches

Geomorphological characteristics of sandy beaches (e.g. sand compaction, grain size) of sandy beaches are important determinants of burrowing preference and behavior (Lim, Yong, & Tantichodok, 2011; Dixon, Peters, & Townsend, 2015). Thus, we measured the sand compaction rate and the sand grain size. Sand compaction rate was measured using a pocket penetrometer with the adapter foot (AMS, E-280) on the first day of the longevity study. We measured the sand compaction rate three times in each quadrat and used the mean value. For the sand grain analysis, we collected three 500 g sand samples from each study sites. These were dried at 70 ⁰C for 24 and were then passed through a series of sieves of a variety of sizes (Folk, 1980).

Burrow longevity and burrow density are inversely related to each other in ghost crab populations (Hughes, 1966). Thus, we measured the burrow density by observing the number of burrows in the quadrats on the first day of the longevity experiment.

**Appendix 3:** Analysis of burrow volume

To obtain the volume of the burrows, we poured a 2:1 mixture of plaster of Paris and freshwater into the burrows (Chan, Chan, & Leung, 2006). After about 30 minutes, we excavated the casts using a shovel and tagged them for later measurement. In the laboratory, a small subsample of the same plaster mixture was prepared in a container whose volume was known. All casts and this subsample were then dried to constant weight at 70 ⁰C. The density of the mixture was determined by using the weight of the subsample of known volume. We then weighed the burrow casts and obtained the volume of each by dividing this density by the mass of each burrow.

**Appendix 4:** Geomorphological characteristics and burrow density

**Table S4.** Geo-morphological variables and burrow density of ghost crabs on the study sites. In the Table, P represents the pristine sites, MI represents the moderately impacted sites, HI represents the highly impacted sites by people, and HV represents the highly impacted sites by people and vehicles.

| Site | Latitude  Longitude | Impact types | Sand compaction (kg cm^-2^) | Sand grain size (mm) | Burrow density (indv. m^-2^) |
| --- | --- | --- | --- | --- | --- |
| Waties Island 1 | 33⁰50’52” N  78⁰33’48” W | P | 0.051 | 0.196 | 0.61 |
| Waties Island 2 | 33⁰50’45” N  78⁰34’33” W | P | 0.043 | 0.211 | 0.77 |
| Waties Island 3 | 33⁰50’34” N  78⁰35’22” W | P | 0.047 | 0.234 | 0.73 |
| N. Myrtle Beach 1 | 33⁰49’28” N  78⁰38’55” W | HV | 0.16 | 0.283 | 0.146 |
| N. Myrtle Beach 2 | 33⁰48’50” N  78⁰40’49” W | HV | 0.148 | 0.261 | 0.126 |
| Myrtle Beach 1 | 33⁰42’11” N  78⁰51’47” W | HV | 0.158 | 0.362 | 0.106 |
| Myrtle Beach 2 | 33⁰40’01” N  78⁰54’21” W | HV | 0.174 | 0.331 | 0.12 |
| Garden City Beach | 33⁰34’13” N  79⁰00’11” W | HV | 0.136 | 0.318 | 0.233 |
| Pawley’s Island 1 | 33⁰26’19” N  79⁰06’53” W | MI | 0.089 | 0.251 | 0.25 |
| Pawley’s Island 2 | 33⁰25’49” N  79⁰07’12” W | MI | 0.083 | 0.233 | 0.228 |
| Debidue Island 1 | 33⁰21’15” N  79⁰09’08” W | P | 0.071 | 0.436 | 0.62 |
| Debidue Island 2 | 33⁰20’09” N  79⁰09’28” W | P | 0.089 | 0.384 | 0.71 |
| Isle of Palm 2–1 | 32⁰47’49” N  79⁰45’09” W | HI | 0.16 | 0.234 | 0.246 |
| Isle of Palm 2-2 | 32⁰47’24” N  79⁰46’19” W | HI | 0.11 | 0.211 | 0.193 |
| Isle of Palm 1-1 | 32⁰46’29” N  79⁰48’28” W | MI | 0.094 | 0.208 | 0.392 |
| Isle of Palm 1-2 | 32⁰46’33” N  79⁰48’39” W | MI | 0.11 | 0.227 | 0.426 |
| Sullivan’s Island 1 | 32⁰45’43” N  79⁰49’32” W | HI | 0.147 | 0.294 | 0.22 |
| Sullivan’s Island 2 | 32⁰45’23” N  79⁰50’24” W | HI | 0.131 | 0.269 | 0.186 |
| Folly Beach | 32⁰39’33” N  79⁰55’36” W | MI | 0.096 | 0.304 | 0.26 |
| Burkes Beach | 32⁰11’51” N  80⁰41’28” W | HI | 0.097 | 0.329 | 0.206 |

**References**

Chan, B.K.K., Chan, K.K.Y., & Leung, P.C.M. (2006). Burrow architecture of ghost crab *Ocypode ceratophthalma* on a sandy shore in Hong Kong. Hydrobiologia, 560(1): 43–49. https://doi.org/10.1007/s10750-005-1088-2

Dixon, R.W., Peters, S.L., & Townsend, C.G. (2015). Burrowing preferences of Atlantic ghost crab, *Ocypode quadrata*, in relation to sand compaction in Padre Island National Seashore, Texas. Physical Geography, 36(3), 188–201.

Folk, R.L. (1980). *Petrology of sedimentary rocks*. Hemphill Publishing Company.

Hughes, D. A. (1966). Behavioural and ecological investigations of the crab *Ocypode ceratophthalmus* (Crustacea: Ocypodidae). Journal of Zoology, 150, 129–143.

Lim, S.S., Yong, A.Y., & Tantichodok, P. (2011). Comparison of burrow morphology of juvenile and young adult *Ocypode ceratophthalmus* from Sai Kaew, Thailand. Journal of Crustacean Biology 31, 59–65.
